# Individualizing dosing frequency may improve the efficacy of prophylaxis in patients with von Willebrand disease—a WIL-31 subanalysis

**DOI:** 10.1016/j.rpth.2025.103221

**Published:** 2025-10-10

**Authors:** Robert F. Sidonio, Ana Boban, Toshko Lissitchkov, Laszlo Nemes, Vladimir Vdovin, Claudia Djambas Khayat

**Affiliations:** 1 Department of Pediatrics, Emory University School of Medicine, Atlanta, Georgia; 2 Department of Internal Medicine, University Hospital Centre Zagreb, Zagreb, Croatia; 3 University of Zagreb School of Medicine, Zagreb, Croatia; 4 Department of Chemotherapy, Hemotherapy and Hereditary Blood Diseases at Clinical Hematology Clinic, Specialized Hospital for Active Treatment of Hematological Diseases, Sofia, Bulgaria; 5 National Hemophilia Centre and Haemostasis Department, Central Hospital of Northern Pest – Military Hospital, Budapest, Hungary; 6 Morozovskaya Children’s City Clinical Hospital, Moscow, Russia; 7 Department of Pediatrics, Hotel Dieu de France Hospital, Saint Joseph University, Beirut, Lebanon

**Keywords:** hemostatics, von Willebrand disease, von Willebrand factor concentrate

## Abstract

**Background:**

The efficacy and safety of prophylaxis with wilate, a plasma-derived von Willebrand factor/factor VIII concentrate, was demonstrated in patients with von Willebrand disease of all types in the WIL-31 study.

**Objectives:**

To investigate the reasons for and the impact of changing the dosing frequency during WIL-31.

**Methods:**

Patients received 20 to 40 IU/kg wilate 2 or 3 times weekly for 12 months. Bleeding rates were analyzed in patients who changed dosing regimen during the study.

**Results:**

Of 33 patients in WIL-31, 30 (91%) initiated prophylaxis 2 times weekly. Of these, 7 (21%) patients increased to 3 times weekly dosing during the study. Four (57%) had underlying circumstances leading to the regimen change: undiagnosed gingivitis, multiple comorbidities, severe menarche, and ongoing left/right ankle arthropathy. The median total annualized bleeding rate in these 7 patients improved from 60.6 during on-demand treatment to 24.0 on 2 times weekly (60.4% reduction) and 3.8 on 3 times weekly prophylaxis (overall reduction, 93.7%). The median spontaneous annualized bleeding rate improved from 32.2 during on-demand treatment to 5.8 on 2 times weekly (82.0% reduction) and 2.4 on 3 times weekly prophylaxis (overall reduction, 92.5%). Despite increased dosing, no von Willebrand factor or FVIII accumulation or thrombotic events were observed.

**Conclusion:**

In the WIL-31 study, a small proportion of patients increased the frequency of prophylaxis, which resulted in effective prevention of bleeding. In most cases, there were underlying medical conditions that necessitated the increased dosing frequency, indicating that personalization of prophylaxis may be beneficial in patients with von Willebrand disease.

**Trial registration details:**

NCT04052698; https://clinicaltrials.gov/study/NCT04052698

## Essentials

• Bleed prevention therapy is recommended for people with von Willebrand disease.
• In WIL-31, prophylaxis with wilate helped prevent bleeds in people with all types of von Willebrand disease.
• Some patients in WIL-31 needed more frequent dosing, mostly due to underlying medical conditions.
• Increasing wilate from 2 to 3 times weekly dosing in these patients effectively reduced bleeding rates.

## Graphical abstract

**Figure:**
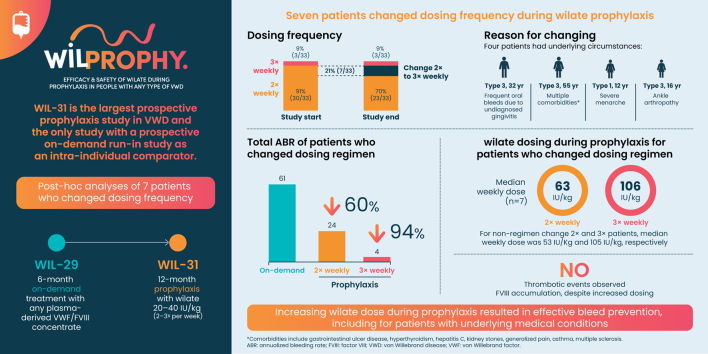

## 1 Introduction

von Willebrand disease (VWD) is the most common inherited bleeding disorder, affecting approximately 1% of the population [1]. VWD is caused by a quantitative or qualitative deficiency in von Willebrand factor (VWF), together with deficiency in factor VIII (FVIII) in many cases, which leads to increased bleeding symptoms and reduced health-related quality of life [2]. Prophylaxis with a VWF concentrate is recommended for people with VWD who have a history of frequent and severe bleeding [3]. Long-term prophylaxis aims to minimize bleeding rates in people with VWD and ultimately improve their health-related quality of life [4]. Although prophylaxis is well-established in hemophilia, it is currently underutilized in VWD [5]. Guidance is not yet well-defined regarding target levels of VWF or FVIII during prophylaxis or the optimal dosing regimen [3]. Establishing optimal dosing regimens are complicated by the heterogenous nature of VWD, which is characterized by multiple subtypes and diverse bleeding symptoms [3].

The phase 3 WIL-31 study was the largest prospective, international, multicenter study of VWF prophylaxis with an intrapatient comparison [6]. WIL-31 demonstrated the efficacy of prophylaxis with wilate, a plasma-derived VWF/FVIII concentrate containing VWF and FVIII in a 1:1 activity ratio, in adults and children (>6 years) with VWD of all types. The primary endpoint of >50% reduction in mean total annualized bleeding rate (TABR) was met, showing a decrease of 84% compared with prior on-demand treatment [6].

Based on the results of WIL-31, wilate was approved for prophylaxis in adults and children (>6 years ) in the United States, with a recommended dosing regimen of 20 to 40 IU/kg, 2 to 3 times weekly [7]. However, individual patients differ in terms of their bleeding phenotype, lifestyle, activity levels, and many other variables [8]. Some patients may therefore require higher levels of hemostatic coverage than others. Here, we report on patients who required an increase in dosing frequency during the WIL-31 study and the impact on bleeding rates.

## 2 Methods

WIL-31 (NCT04052698; WILPROPHY) was a prospective, noncontrolled, international, multicenter phase 3 trial of prophylaxis in patients with severe VWD [6]. Prior to entering the WIL-31 study, all patients had received on-demand treatment with a VWF-containing concentrate during a 6-month, prospective, observational, run-in study (WIL-29). Patients in WIL-31 received regular wilate prophylaxis 2 to 3 times per week at a dose of 20 to 40 IU/kg for 12 months. Each patient’s initial dosing regimen was determined at the physician’s discretion based on their clinical condition. The dosage was to be increased by ∼5 IU/kg in the event of >2 spontaneous bleeding events or 1 major bleeding event within a 30-day period. If >2 spontaneous bleeding events were experienced following a dose increase in patients on a 2 times weekly regimen, then the dosing regimen was to be changed to 3 times weekly. The primary endpoint of WIL-31 was the mean TABR during wilate prophylaxis compared with prior on-demand treatment (ie, during WIL-29). FVIII and VWF activity levels were measured at baseline and at 1, 2, 3, 6, 9 and 12 months of treatment, using the VWF Ristocetin cofactor assay and FVIII one-stage and chromogenic assays. Blood samples were taken within 60 minutes before and 60 ± 5 minutes after a prophylaxis infusion. The study was performed in accordance with the Declaration of Helsinki and the respective local regulations. Fully informed written and signed consent was voluntarily given by patients (or their legal guardians) before any study-related procedures were conducted. For further details on study design, please refer to the primary publication [6]. Post hoc analyses were performed on patients who changed dosing frequency during the WIL-31 study. The following parameters were assessed: median TABR and spontaneous ABR (SABR), dose of wilate (IU/kg) before and after dosing frequency change, and preinfusion and postinfusion plasma levels of FVIII and VWF over time. All statistical analyses are descriptive.

## 3 Results and Discussion

The WIL-31 study evaluated 33 patients with confirmed VWD. Of these, 91% (30 patients) initiated prophylaxis on a 2 times weekly dosing regimen and 9% (3 patients) on a 3 times weekly regimen. Seven patients (21.2%) changed dosing frequency during WIL-31 from 2 times weekly to 3 times weekly (regimen change [RC]), and 26 patients did not change the frequency of dosing throughout the study (non-RC). Data for non-RC patients are presented for comparison and comprise 2 subpopulations: 23 patients who received wilate 2 times weekly (non-RC 2×) and 3 patients who received wilate 3 times weekly (non-RC 3×). Of the 7 RC patients, 2 had type 1, 1 had type 2A, and 4 had type 3 VWD. The median (range) age was 16 (8-55) years for the RC patients. There were no major differences in baseline FVIII or VWF activity levels between the patient groups. Further information on individual RC patients is summarized in the Table.

**Table:** Characteristics of individual patients who increased dosing frequency during WIL-31.

VWD type, age at enrollment	Biological sex	Child-bearing potential	VWD family history	Underlying circumstances	Reason for switching	Comments
Type 3, 32 y	Male	N/A	No	Yes	7 spontaneous bleeds (6 oral, 1 nose) between day 78 and 133 of study; 9 spontaneous oral bleeds in the following 1 mo	Diagnosed with gingivitis, no spontaneous bleeds after dental hygiene consult
Type 3, 55 y	Female	No	No	Yes	12 spontaneous bleeds in 3 mo	Comorbidities include gastrointestinal ulcer disease, hyperthyroidism, hepatitis C, kidney stones, generalized pain, asthma, multiple sclerosis
Type 2A, 46 y	Male	N/A	Yes	No	Increased frequency after 1 spontaneous nosebleed (minor; duration of 1.5 h; successfully treated with 1 dose of wilate) No sufficient decrease in frequency of nose bleeds	All spontaneous bleeds were nosebleeds
Type 1, 12 y	Female	No^a^	No	Yes	Regimen change following first menstruation, which required hospitalization and transfusion	Two heavy menstrual bleeds occurred after regimen change, 1 of which required hospitalization Study ended before impact of the regimen change could be assessed
Type 1, 9 y	Male	N/A	Yes	No	12 spontaneous bleeds within 7 mo	All spontaneous nosebleeds
Type 3, 16 y^b^	Female	No	Yes	Yes	Regimen change following episodes of ankle bleeding that limited activity	Left and right ankle arthropathy. Traumatic bleeds accounted for 12/13 bleeds (nose and joint; 10 traumatic bleeds occurred prior to regimen change and 2 afterwards)
Type 3, 8 y^b^	Male	N/A	No	No	Patient engaged in high-risk physical activities, necessitating regimen change	3 nosebleeds attributed to allergic rhinitis

N/A, not applicable; VWD, von Willebrand disease.

a At time of enrollment.

b Based on bleeding history; initiating prophylaxis on a 3 times weekly regimen was recommended, but patients did not agree to this initial regimen.

**Figure 1:**
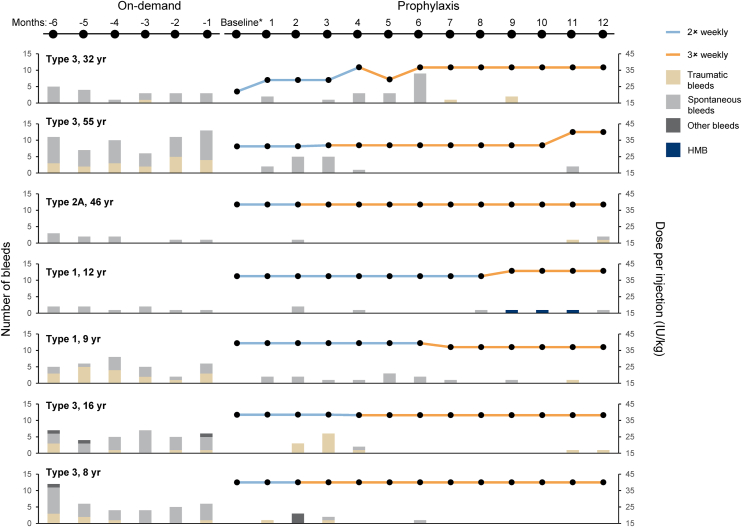
Bleeds during on-demand treatment and prophylaxis, and prophylactic dose per month for the 7 patients who increased dosing frequency during WIL-31. ∗Regimen change did not always occur on first day of month. HMD, heavy menstrual bleeding.

RC patients received 2 times weekly and 3 times weekly prophylaxis for a mean (SD) duration of 4.7 (2.9) and 7.5 (2.9) months, respectively. The median dose per injection in RC and non-RC patients was 37.9 IU/kg and 27.9 IU/kg, respectively. The median (range) weekly dose over the study period was 88.4 (52.6-113.7) IU/kg for RC patients, 52.6 (28.2-72.1) IU/kg for non-RC 2× patients and 105.4 (98.4-112.3) IU/kg for non-RC 3× patients. For RC patients, median (range) weekly dose when receiving 2 times weekly prophylaxis was 62.6 (53.5-89.1) IU/kg, compared with 105.7 (37.9-118.9) IU/kg when receiving 3 times weekly prophylaxis. Individual durations of prophylaxis and doses per injection for each of the 7 RC patients are summarized in Figure 1.

**Figure 2:**
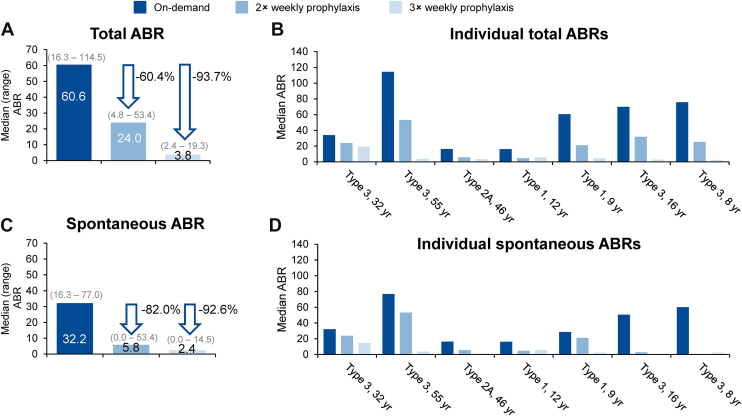
Total and spontaneous ABRs during on-demand treatment, 2 times weekly prophylaxis and 3 times weekly prophylaxis for the 7 patients who increased dosing frequency during WIL-31. ABR, annualized bleeding rate.

RC patients experienced improvements in TABR while on 2 times weekly prophylaxis; the median (range) TABR improved from 60.6 (16.3-114.5) during on-demand treatment to 24.0 (4.8-53.4) on 2 times weekly prophylaxis, a reduction of 60.4%. Changing to a 3 times weekly prophylactic regimen further improved median (range) TABR to 3.8 (2.4-19.3), a reduction of 93.7% vs on-demand (Figure 2A).

During on-demand treatment, RC patients experienced 215 bleeds (72% spontaneous, 71% minor) with a median SABR of 32.2 (range: 16.3-77.0; Figure 2C). During 2 times weekly prophylaxis, there were 54 bleeds (72% spontaneous, 95% minor) with a median SABR of 5.8 (0.0-53.4), corresponding to a reduction of 82.0% compared with the on-demand period. After the dosing frequency was increased, RC patients experienced 25 bleeds (68% spontaneous, 100% minor) with a median SABR of 2.4 (0.0-14.5), a reduction of 92.6% vs on-demand. Individual TABRs and SABRs for the 7 RC patients are shown in Figure 2B, D. All patients experienced improvements in TABR and SABR while on 2 times weekly prophylaxis vs on-demand treatment, and even greater improvements were observed after the dosing was increased to 3 times weekly.

**Figure 3:**
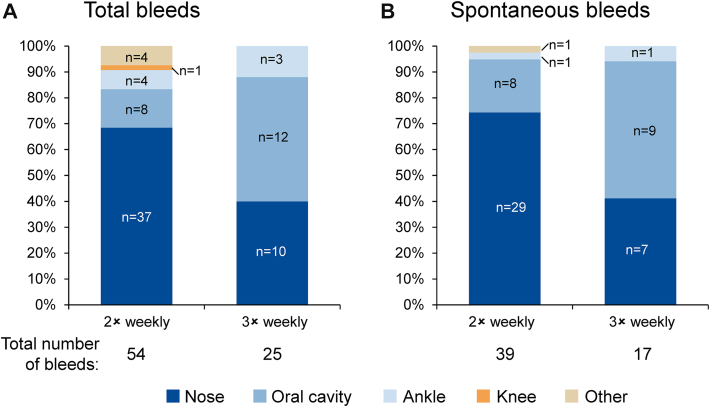
Bleed sites of total and spontaneous bleeds on 2 times vs 3 times weekly prophylaxis for the 7 patients who increased dosing frequency during WIL-31.

During WIL-31, RC patients experienced bleeds at the following sites: nose, oral cavity, ankle joint, and knee joint (Figure 3A). Nosebleeds were the most common bleed, making up 64.3% of all spontaneous bleeds in RC patients over the study period (Figure 3B). All nose bleeds were minor. In RC patients, 2 times weekly prophylaxis reduced the median nosebleed SABR by 70.8% compared with on-demand treatment, from 16.4 (range: 0.0-73.1) to 4.8 (range: 0.0-49.0). Increasing dosing frequency to 3 times weekly reduced the median nosebleed SABR by 86.6% vs on-demand treatment to 2.2 (range: 0.0-5.7).

Despite the increases in dosing frequency, no accumulation of VWF or FVIII was observed in any patient (Supplementary Figures 1-3). No thrombotic events were observed in either RC or non-RC patients.

Differences in patients’ lifestyles, activity levels, and other variables lead to differing requirements for hemostatic cover [8]. Of the 7 patients who increased dosing frequency in WIL-31, 4 had underlying circumstances: undiagnosed gingivitis, multiple comorbidities, severe menarche, or ongoing left/right ankle arthropathy (Table). For some cases, such as the patient with gingivitis, bleeding was improved once the underlying cause was addressed, illustrating the need for identifying and managing factors that may influence the requirement for hemostatic cover, alongside adapting prophylactic regimens. The patients described here presented with unusual and complex cases, and their increases in dosing frequency deviated from study protocol. This highlights the importance of dosing flexibility to provide increased hemostatic cover when required.

In WIL-31, dosing was determined based on the physician’s discretion, depending on the patient’s condition. The weekly dose of wilate during WIL-31 was low, even after increasing the dosing frequency: median of 88.4 IU/kg for RC patients and 52.6 IU/kg for non-RC 2× patients. For comparison, during a similar study of prophylaxis in patients with VWD with recombinant VWF, the median weekly dose was 95.6 IU/kg [9]. Despite this lower weekly dose in WIL-31, a 2 times weekly dosing regimen was effective for bleed control in most patients, and the flexibility to increase injection dose or dose frequency enabled physicians to provide flexible hemostatic cover in individuals with specific needs. In those with hemophilia A, the target during prophylaxis is generally a FVIII trough level of >1% to provide optimal hemostatic cover [10]. However, increasing interest in personalized treatment has led to recommendations for target plasma levels based on individual factors, including patient bleeding phenotype, activity level, prior arthropathy, and comorbidities [8]. In VWD, tailoring target factor levels based on individual patient needs is also recommended, but suggested targets during prophylaxis are not yet determined [3].

Early identification of individuals who may require more frequent hemostatic cover and understanding which variables contribute to this are crucial steps in individualizing prophylactic regimens and improving bleeding symptom management. Population pharmacokinetic (PK) models are used to guide individualized prophylactic regimens in people with hemophilia A [8]. In people with VWD, VWF and FVIII levels can be hard to predict after dosing, with some patients experiencing lower than target activity levels without a clear cause [11]. This individual variation in the PKs of VWF/FVIII concentrates may explain why some patients require more frequent dosing or have better outcomes on 2 or 3 times weekly dosing [11]. PK models for people with VWD that assess the interactions between VWF activity and FVIII levels have been developed and are being investigated [12,13]. Additional studies are needed to further explore how this method could be used to help guide prophylactic regimens in people with VWD.

Another variable that can influence dosing requirements is age. Younger individuals, who have not experienced the long-term impact of repeated bleeding with on-demand treatment, may have different dosing requirements than older individuals. Those who start prophylaxis at a younger age may experience less long-term joint damage and require less frequent infusions later in life to prevent joint bleeds [14]. Prophylaxis with wilate was efficacious and well-tolerated in children (aged 6-11 years) and adolescents (aged 12-16 years) with all types of VWD [15]. The ongoing WIL-33 trial (NCT04953884 [16]) is investigating the use of wilate prophylaxis in children aged <6 years. Results from this study will provide more insights into the efficacy of wilate prophylaxis in preventing bleeding events and provide crucial data about dosing in children aged <6 years. Limitations of the WIL-31 study include that it was open-label, uncontrolled, and comprised mostly Caucasian patients, and no patients had type 2N VWD. Additionally, these analyses were post hoc, and due to the small number of patients who changed dosing regimen, sample sizes were low.

## 4 Conclusions

These analyses from the WIL-31 study show that in patients requiring greater hemostatic coverage, increasing from a 2 to 3 times weekly prophylactic regimen with wilate was effective in reducing bleeding rates in patients with VWD. For those patients who required an increase in dosing frequency, most had underlying circumstances that led to the change. Patients’ individual needs should be considered when deciding on personalized dosing regimens.
