# Supplementary material for: Individualizing dosing frequency may improve the efficacy of prophylaxis in patients with von Willebrand disease—a WIL-31 subanalysis
Source: Res Pract Thromb Haemost. 2025 Oct 10;9(7):103221. doi: 10.1016/j.rpth.2025.103221 (PMC12634286; doi:10.1016/j.rpth.2025.103221)
Supplement: Supplementary Material [file mmc1.docx]

**Supplementary Material**

**Supplementary Figure 1: Individual FVIII activity (FVIII:C) levels (pre- and post-injection) during 12 months of wilate prophylaxis for the 7 patients who increased dosing frequency.**

**
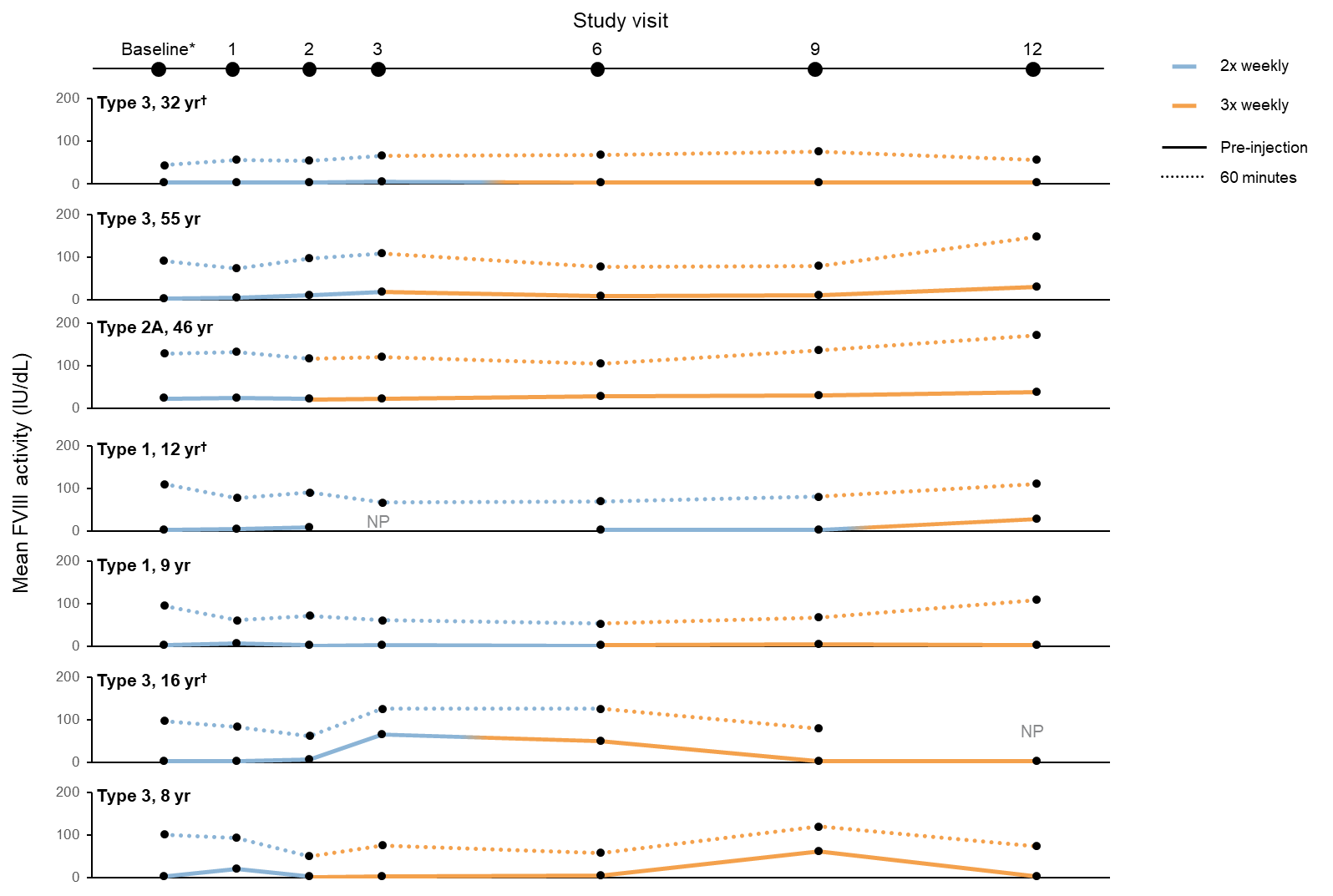
**

*RC did not always occur on first day of month. ^†^Dosing change occurred between study visits where activity was tested. FVIII: factor VIII; FVIII:C: FVIII chromogenic assay; NP: test not performed by Central Laboratory.**Supplementary Figure 2: Individual FVIII activity (FVIII:OS) levels (pre- and post-injection) during 12 months of wilate prophylaxis for the 7 patients who increased dosing frequency.**

**
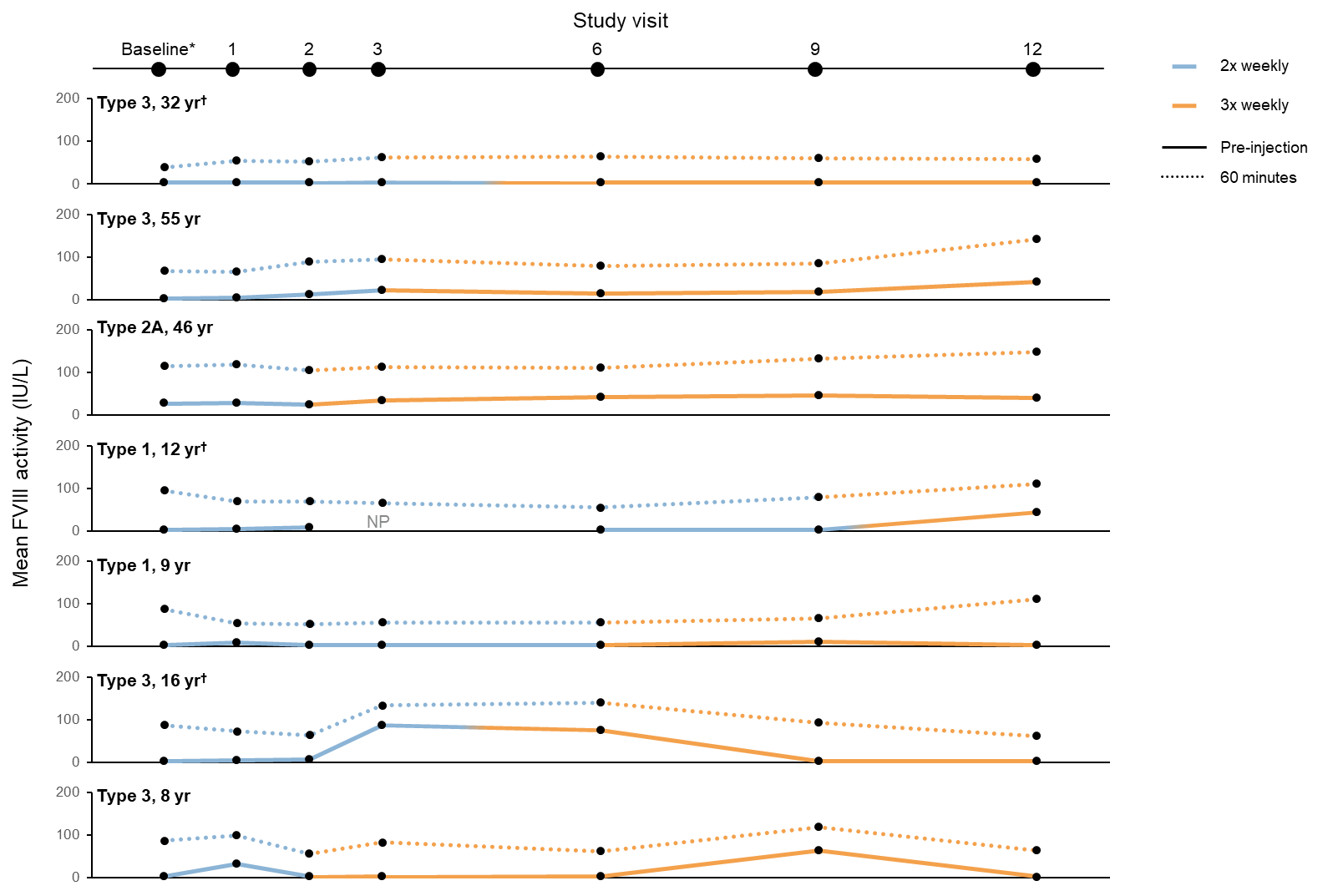
**

*RC did not always occur on first day of month. ^†^Dosing change occurred between study visits where activity was tested. FVIII: factor VIII; FVIII:OS: FVIII one-stage assay; NP: test not performed by Central Laboratory.

**Supplementary Figure 3: Individual VWF activity levels (pre- and post-injection) during 12 months of wilate prophylaxis for the 7 patients who increased dosing frequency.**

**
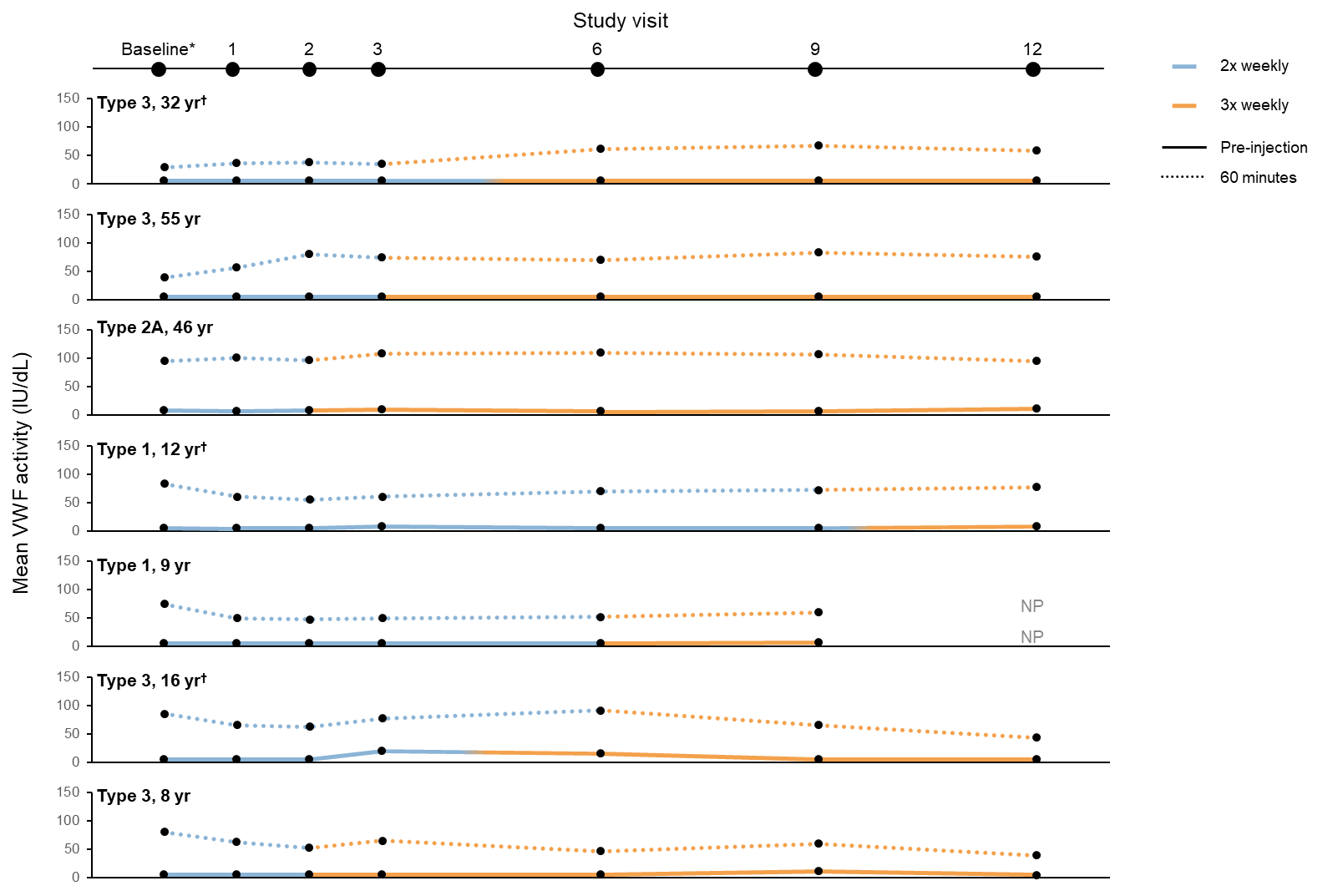
**

*RC did not always occur on first day of month. ^†^Dosing change occurred between study visits where activity was tested. NP: test not performed by Central Laboratory; VWF: von Willebrand factor.
